# Gender-Related Differences in Trimethylamine and Oxidative Blood Biomarkers in Cardiovascular Disease Patients

**DOI:** 10.3390/biomedicines8080238

**Published:** 2020-07-23

**Authors:** Laura Bordoni, Donatella Fedeli, Marco Piangerelli, Iwona Pelikant-Malecka, Adrianna Radulska, Joanna J. Samulak, Angelika K. Sawicka, Lukasz Lewicki, Leszek Kalinowski, Robert A. Olek, Rosita Gabbianelli

**Affiliations:** 1 Unit of Molecular Biology, School of Pharmacy, University of Camerino, 62032 Camerino, Italy; laura.bordoni@unicam.it (L.B.); donatella.fedeli@unicam.it (D.F.); 2 Computer Science Division and Mathematics Division, School of Science and Technology, University of Camerino, 62032 Camerino, Italy; Marco.piangerelli@unicam.it; 3 Department of Medical Laboratory Diagnostics, Medical University of Gdansk, 80-211 Gdansk, Poland; iwpe-ma@gumed.edu.pl (I.P.-M.); adrianna.radulska@gumed.edu.pl (A.R.); leszek.kalinowski@gumed.edu.pl (L.K.); 4 Biobanking and Biomolecular Resources Research Infrastructure Poland (BBMRI.PL), 80-211 Gdansk, Poland; 5 Doctoral School for Physical Culture Sciences, 80-336 Gdansk, Poland; joanna.samulak@awf.gda.pl (J.J.S.); angelika.sawicka@awf.gda.pl (A.K.S.); 6 Department of Human Physiology, Faculty of Health Sciences, Medical University of Gdansk, 80-210 Gdansk, Poland; 7 University Center for Cardiology, Gdansk, Debinki 2, 80-211 Gdansk, Poland; luklewicki@gmail.com; 8 Gdansk University of Technology, Narutowicza 11/12, 80-233 Gdansk, Poland; 9 Department of Athletics, Strength and Conditioning, Poznan University of Physical Education, 61-871 Poznan, Poland

**Keywords:** gender, membrane erythrocyte, hydroperoxides, biomarker, DPPP, DPH, TMA, cardiovascular disease, data analysis, precision–recall

## Abstract

Gender differences in the burden of cardiovascular disease (CVD) have been observed worldwide. In this study, plasmatic levels of trimethylamine (TMA) and blood oxidative biomarkers have been evaluated in 358 men (89 controls and 269 CVD patients) and 189 women (64 control and 125 CVD patients). The fluorescence technique was applied to determine erythrocyte membrane fluidity using 1,6-diphenyl-1,3,5-hexatriene (DPH) and Laurdan, while lipid hydroperoxides were assessed by diphenyl−1-pyrenylphosphine (DPPP). Results show that levels of plasmatic TMA were higher in healthy men with respect to healthy women (*p* = 0.0001). Significantly lower TMA was observed in male CVD patients (0.609 ± 0.104 μM) compared to healthy male controls (0.680 ± 0.118 μM) (*p* < 0.001), while higher levels of TMA were measured in female CVD patients (0.595 ± 0.115 μM) with respect to female controls (0.529 ± 0.073 μM) (*p* < 0.001). DPPP was significantly higher in healthy control men than in women (*p* < 0.001). Male CVD patients displayed a lower value of DPPP (2777 ± 1924) compared to healthy controls (5528 ± 2222) (*p* < 0.001), while no significant changes were measured in females with or without CVD (*p* > 0.05). Membrane fluidity was significantly higher (*p* < 0.001) in the hydrophobic bilayer only in control male subjects. In conclusion, gender differences were observed in blood oxidative biomarkers, and DPPP value might be suggested as a biomarker predictive of CVD only in men.

## 1. Introduction

Cardiovascular disease (CVD) represents one of the primary causes of death worldwide. Data from the World Health Organization (WHO) identify CVD responsible for 37% of all deaths in the European Union (40% and 34% of all deaths in females and males, respectively). Ischemic heart disease accounts for 20% of CVD deaths in females and 19% in males. Stroke is the second most common cause of CVD deaths, accounting for 13% of all CVD deaths in females and 9% in males [1].

Genetics, gender, ethnicity, lifestyle, and socioeconomical position contribute to outline cardiovascular health from a prenatal period of life. Diseases like diabetes, hypertension, obesity, and unhealthy diets are major risk factors [2]. Genes on sex chromosomes can influence the genes in autosomes and sex steroids testosterone and estrogen; variations of androgen receptor, whose locus is on chromosome X, determine sex differences in cardiovascular regulatory mechanisms [3].

CVD can be influenced by early life risk factors; indeed, an association between low body weight at birth and an increased risk of developing CVD later in life has been observed in both genders [4,5]. Maternal low protein intake and/or high fat diet increase the risk of developing non-communicable diseases; time-dependent malnutrition during prenatal life modulates the epigenome differently, leading to CVD or other diseases (i.e., obesity, glucose intolerance) in adult age, according to biological sex [6,7].

During recent years, several studies have highlighted differences in the incidence of heart disease in women and men [8,9,10,11,12,13,14]. The prevalence of CVD is higher in males than in females [8]. Women develop CVD later in life, with a higher risk of stroke [9]. A different number of deaths due to CVD is also reported [10,11].

The increased incidence of death in women due to CVD seems to be associated with atypical symptoms [10] and different distributions of risk factors [11]. Diabetes and dyslipidemia have a higher impact in females than in males [11]. Women in their premenopausal life are more protected from CVD than men by estrogens, so their first myocardial infarction occurs when they are about ten years older than men. Epidemiological analysis in the period 1980–2010 reveals that CHD incidence was more reduced in men than in women; however, mortality due to CHD was higher in men than in women until old age [9]. A large case-control study (involving 24,767 people from 262 centers in 52 countries) on the association between modifiable risk factors (i.e., diet, alcohol intake, smoking, physical inactivity, psychosocial stress) and CHD incidence shows a higher impact of social determinants in women than in men [15]. Thus, better identification of sex differences in CVD is of crucial significance to develop additional strategies to prevent and treat more efficiently CVD in women and men.

In this context, oxidative stress represents a key process depending on several aspects (i.e., genetics, lifestyle, environment, etc.) that can affect numerous cell functions. Active metabolites are produced following redox reactions; e.g., the oxidation of trimethylamine (TMA) to trimethylamine N-oxide (TMAO) [16], which can be monitored due to its involvement in inflammation, atherosclerosis development, and impact on visceral adiposity [17,18,19,20]. Moreover, at both the plasma membrane level and lipoproteins, lipid oxidation leads to hydroperoxides production, which significantly contributes to the oxidation of TMA into TMAO, as it has been observed from in vitro and ex vivo studies [21,22]; then, since TMAO is a reactive molecule, it might promote foam cells formation [23]. The crosstalk between lipid peroxidation and TMA metabolism might include gender differences that could provide further insights on CVD; hence, the collection of evidence on this aspect could promote a gender approach to modulate CVD risk.

To identify biomarkers useful to characterizing gender differences in CVD, the present study assesses several blood biomarkers: the plasma TMA, the erythrocyte plasma membrane fluidity, and lipid hydroperoxides are measured in 358 men (89 controls and 269 CVD patients) and 189 women (64 controls and 125 CVD patients). A different profile in controls and CVD subjects has been identified according to gender; a direct correlation between plasma TMA level and erythrocyte lipid hydroperoxides measured by the DPPP probe has been measured in men, while DPH fluorescence anisotropy was inversely correlated to TMA and DPPP.

## 2. Experimental Section

### 2.1. Subjects

In total, 358 male (89 controls and 269 CVD patients) and 189 females (64 controls and 125 CVD patients) were recruited in Wejherowo Cardiovascular Center. Inclusion criteria for CVD patients were the diagnosis of elective or urgent coronary angiography, established according to the Third Universal Definition of Myocardial Infarction, and 2015 European Society of Cardiology guidelines [24,25]. The control group was recruited amongst the subjects without a self-reported medical history of CVD (Table 1). The present study was approved by the Regional Bioethical Committee in Gdansk (KB-27/16, 28 December 2016 and extended KB 32-17, 16 November 2017). Informed consent was obtained from all subjects.

### 2.2. Samples Collection

Blood samples were collected in EDTA-containing tubes; plasma samples obtained by centrifugation at 1300× *g* for 10 min at 18–25 °C were kept frozen at −80 °C until TMA analysis. Erythrocytes were kept frozen at −80 °C and used for membrane isolation.

### 2.3. Plasma TMA and TMAO

Plasma TMA and TMAO were determined by the Ultra High Performance Liquid Chromatography (UHPLC) tandem mass spectrometry method, based on methods described previously [26,27]. UHPLC separation was performed on an XBridge ^®^ HILIC 3.5 μm (3.0 × 50 mm, Waters, MA, USA) column on a NEXERA Shimadzu UHPLC system coupled with QT4500 SCIEX. Trimethyl-d9-amine HCl (d9-TMA, C/D/N ISOTOPES INC., Quebec, Canda) was used as an internal standard. The 3 μM of d9-TMA working solution of internal standard (ISWS) was prepared in methanol/acetonitrile (15:85) and 0.1% formic acid (*v/v*) (Merck KGaA, Darmstadt, Germany). Calibration samples, QC, and plasma samples were prepared by the addition 100 μL of cold ISWS to 50 μL of each sample type. All samples were vortexed and kept on ice for 15 min for protein precipitation. Centrifuged samples (14,000 rpm, 4 °C, for 20 min.) were divided into two parts: without dilution, which were used for the analysis of TMA concentration and diluted (5:95 of ISWS) for analysis of TMAO. The mobile phase was 70% of acetonitrile with 0.1% formic acid (*v/v*) and 30% of 15 mmol/L ammonium formate with 0.1% formic acid (*v/v*) (Merck KGaA, Darmstadt, Germany) at a flow rate of 0.4/min. The mass spectrometer was operated in multiple-reaction monitoring (MRM)-positive electrospray ionization (ESI+). The mass spectrometer optimized settings were as follows: IonSpray Voltage = 5.5 kV, source temperature = 300 °C, collision gas = 8, and curtain gas = 30.0. Ion transitions used for quantitation: *m*/*z* 60.1 → 44.0 for TMA, *m*/*z* 76.1 → 59.0 for TMAO and *m*/*z* 69.1 → 49.1 for d9-TMA. Calibration curves ranges were from 0.1 to 30 μM and 0.3 to 30 μM for TMA and TMAO, respectively. The limits of quantification were 0.1 μM for TMA and 0.3 μM for TMAO.

### 2.4. Erythrocyte Plasma Membrane Preparation

Erythrocyte membranes were obtained by progressive hemolysis; cells were incubated in 5 mM Na_2_HPO_4_, pH 7.4 at 4 °C for 20 min and centrifuged at 9000× *g* for 15 min at 4 °C. The additional steps of incubation with 2.5 and 1.25 mM Na_2_HPO_4_ at pH 7.4 and centrifugation were done to obtain white membranes.

### 2.5. Erythrocyte Membrane Fluidity

Membrane fluidity was measured on erythrocyte membrane using 1,6-Diphenyl−1,3,5-hexatriene (DPH) (Molecular Probes, Eugene, OR, USA) and 6-lauroyl−2-dimethylaminonaphthalene (Laurdan) (Molecular Probes, Eugene, OR, USA).

A Hitachi 4500 spectrofluorometer was used for fluorescence measurements.

All samples were normalized to the final protein concentration of 0.4 mg/mL, determined using the Pierce BCA Protein Assay Kit (Thermo Scientific, Waltham, MA, USA).

Steady-state fluorescence anisotropy (r) of DPH was carried out in 1 mL of sodium phosphate buffer (2.5 mM, pH 7.4), containing 0.4 mg of the plasma membrane and DPH at the final concentration of 1 µM after 1 h of incubation at 37 °C using excitation and emission wavelengths of 360 and 430 nm, respectively, according to the equation [28]:

r = (I| | − I⊥g)/(I| | + 2I⊥g)(1)

where g is the grating correction factor for the optical system, obtained from the ratio of the perpendicular and the parallel polarized emission components when the excitation light is polarized in the horizontal plane, and I_||_ and I_⊥_ are the intensities measured with the polarization plane parallel and perpendicular to that of the exciting beam.

Generalized polarization of Laurdan (GP340) was analyzed in 1 mL of sodium phosphate buffer (2.5 mM, pH 7.4), containing 0.4 mg of the plasma membrane and Laurdan at the final concentration of 1 µM after 5 min of incubation at 37 °C using an excitation wavelength of 340 and emission of 440 and 490 nm, respectively, according to Parasassi’s equation [29]:

GP340 = (I_B_ − I_R_)/(I_B_ + I_R_) (2)

where I_B_ and I_R_ are the intensities at the blue (440 nm) and red (490 nm) edges of the emission spectrum and correspond to the fluorescence emission maximum in the gel and liquid crystalline phases of the bilayer, respectively [30].

### 2.6. DPPP Assay

Erythrocyte membrane lipid hydroperoxides were detected using a membrane-localized fluorescent probe Diphenyl−1-pyrenylphosphine (DPPP) (Cayman Chemical Co., Ann Arbor, MI, USA), that reacts specifically with hydroperoxides and becomes highly fluorescent when oxidized [22]. Lipid hydroperoxide formation occurs considerably in plasma membranes from unsaturated fatty acids during oxidative stress [31,32]; the measure of lipid hydroperoxides represents a crucial step of the propagative lipid peroxidation, which affects membrane and protein conformation with important outcomes on their functionality [32]. DPPP represents a sensitive and reproducible quantitative method of total lipid hydroperoxide analysis [33].

Membrane samples, normalized to the final protein concentration of 0.4 mg/mL, were incubated with 1 μM DPPP in 1 mL PBS at 37 °C for 5 min in the dark. The fluorescence intensity of the samples was measured using 351 and 380 nm as excitation and emission wavelengths, respectively. Results are expressed as fluorescence intensity (arbitrary units, a.u.).

### 2.7. Statistical Analysis

Shapiro–Wilk was used to test the normality of data distribution. The Kruskal–Wallis test and Spearman correlation were used to determine significant differences among analyzed variables and correlations, respectively. Precision–recall curves were calculated using the Scikit-learn library for Python. They were used for testing the power of the logistic regression model based on DPPP and DPH for males and females, respectively. If not differently specified, statistical analyses were performed using the SPSS package for Windows, v.20.0 (SPSS Inc., Chicago, IL, USA).

## 3. Results

### 3.1. Descriptive Statistics

Of the 547 recruited subjects, 358 were male and 189 females. Within the male group, 89 were healthy controls, while 269 were CVD patients. The male controls’ and CVD patients’ mean ages were 62.6 ± 8.5 and 65.0 ± 12.0, respectively. Within the female group, 64 women were healthy controls, while 125 were CVD patients. The female controls’ and CVD patients’ mean ages were 66.8 ± 6.7 and 69.5 ±10.3, respectively.

### 3.2. Gender Differences in the Plasma TMA Level

**Figure 1:**
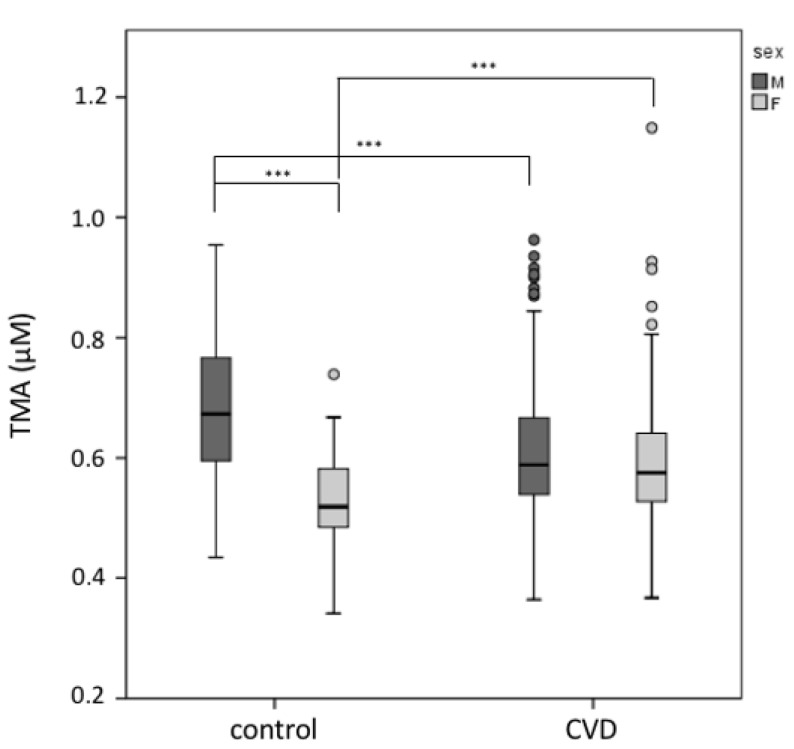
Plasma TMA level in males and females. The male control group displays higher plasma TMA levels compared to female controls. Male CVD patients show a lower TMA value with respect to healthy controls of the same gender, while an opposite result was observed in the female groups. Male controls (*n* = 89) and male CVD patients (*n* = 269). Female controls (*n* = 64) and female CVD patients (*n* = 125), *** *p* < 0.001.

Plasma TMA levels were significantly different in the two genders, i.e., healthy control men show higher levels of TMA (0.680 ± 0.118 μM) than women (0.529 ± 0.073) (*p* = 0.0001). Plasma TMA levels were significantly lower in male CVD patients compared to controls (Figure 1) (*p* < 0.001). At the same time, a higher TMA level in female CVD subjects with respect to healthy controls of the same gender was observed (Figure 1) (*p* < 0.001).

### 3.3. Gender Differences in the Erythrocyte Plasma Membrane Fluidity

**Figure 2:**
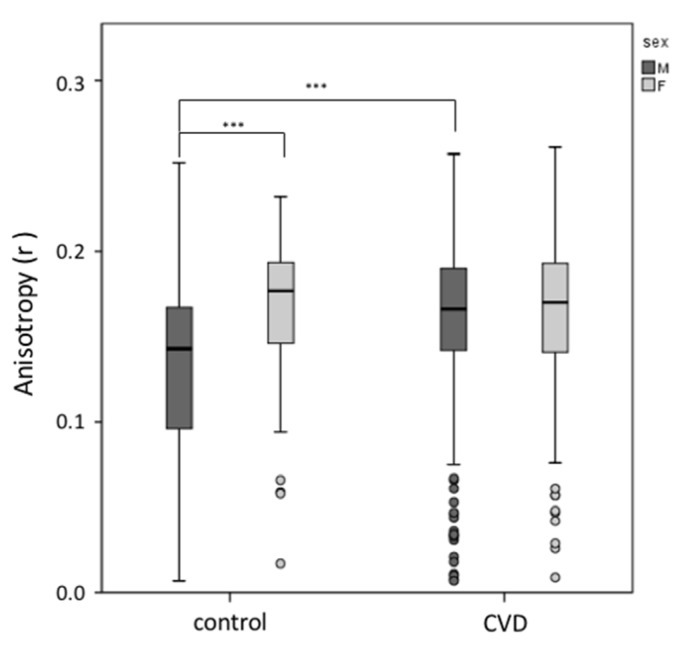
DPH fluorescence anisotropy (r) in plasma membrane erythrocytes from males and females. The measured membrane fluidity was higher in male controls with respect to female controls. Male CVD patients have a lower DPH membrane fluidity with respect to healthy controls of the same gender. Male controls (*n* = 89) and male CVD patients (*n* = 269), *** *p* < 0.001. Female controls (*n* = 64) and female CVD patients (*n* = 125).

Erythrocyte plasma membrane fluidity was analyzed using fluorescent probes that localize at different depths of the bilayer. The analysis of the hydrophobic region of the bilayer by DPH showed significant changes in fluorescence anisotropy (r) in controls: male subjects have a lower value of anisotropy (r = 0.129 ± 0.558) compared to females (r = 0.166 ± 0.425) (*p* < 0.001). Male CVD patients present a higher value of anisotropy with respect to controls (Figure 2) (*p* < 0.001), while no changes were observed in female CVD subjects with respect to healthy controls of the same gender (Figure 2) (*p* > 0.05).

The analysis of the hydrophilic–hydrophobic region by Laurdan showed no changes in generalized polarization of Laurdan between males and females, as well as in healthy/unhealthy subjects.

### 3.4. Gender Differences in the Erythrocyte Lipid Hydroperoxide Level (DPPP)

**Figure 3:**
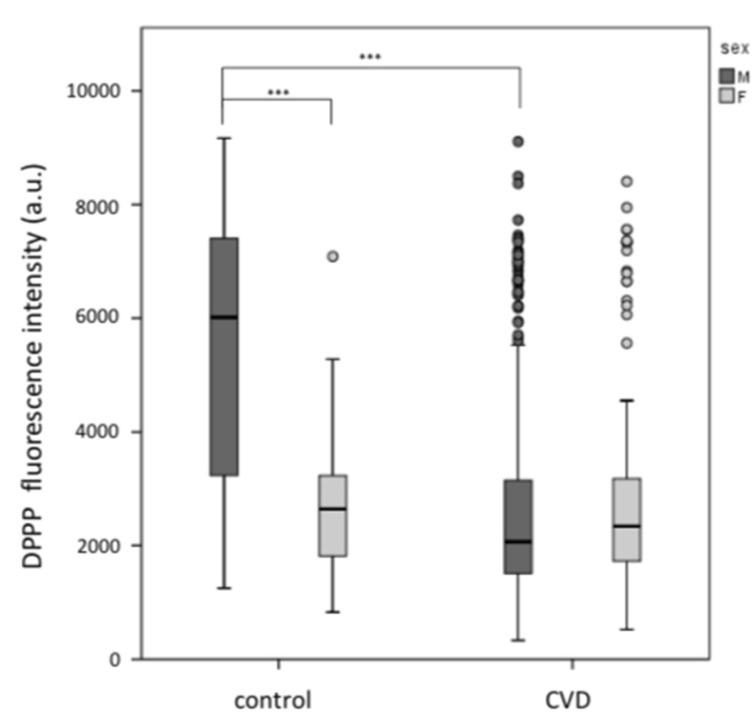
Lipid hydroperoxides (DPPP) in erythrocytes from male and female subjects. Lipid hydroperoxides were higher in the male control group with respect to female controls. Male CVD patients have a lower hydroperoxides level with respect to healthy controls of the same gender. Male controls (*n* = 89) and male CVD patients (*n* = 269), *** *p* < 0.001. Female controls (*n* = 64) and female CVD patients (*n* = 125). Results are expressed as fluorescence intensity (arbitrary units, a.u.).

**Figure 4:**
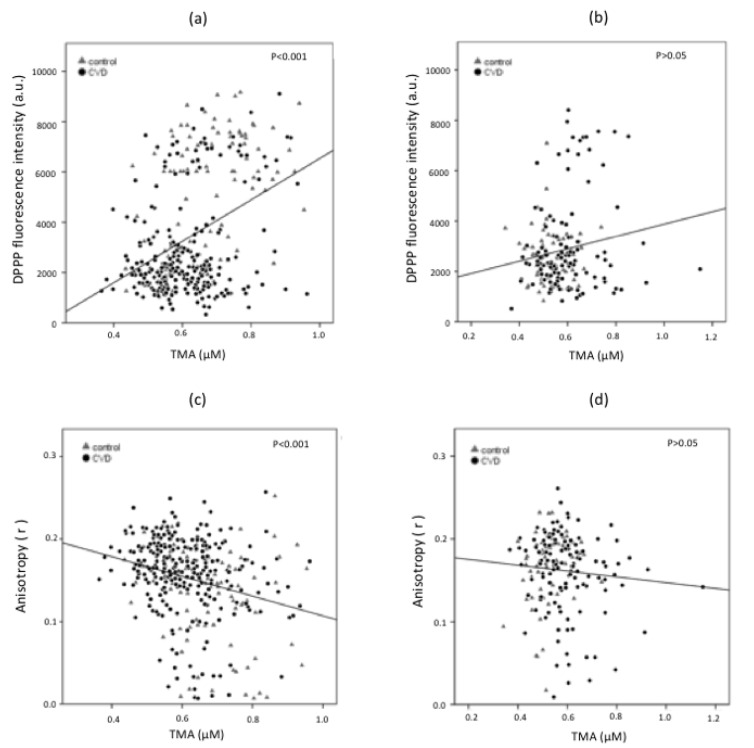
Correlations between plasma TMA levels and DPPP in males (**a**) or females (**b**), and DPH anisotropy (r), in the male (**c**) or female (**d**) groups. Only in the male group, erythrocyte membrane lipid hydroperoxide (DPPP fluorescence intensity, a.u.) correlates positively, and membrane fluidity (anisotropy, r) inversely with plasma TMA.

Lipid hydroperoxide levels were measured in erythrocyte plasma membrane using the fluorescent probe DPPP. DPPP fluorescence was significantly different between healthy males and females, with men displaying higher values (5528 ± 2222) than women (2616 ± 1065) (*p* < 0.001). Male CVD patients show lower values of DPPP compared to healthy controls of the same gender (Figure 3) (*p* < 0.001), while no changes in females with or without CVD were measured (Figure 3) (*p* > 0.05). Interestingly, the DPPP was directly related to TMA levels (Spearman correlation = 0.317; *p* < 0.001) in the male group (Figure 4), but not in the female group (Spearman correlation = 0.067; *p* = 0.361) (Figure 4). On the other hand, TMA showed a negative correlation with DPH in males (*p* < 0.001) (Figure 4), but not in females (*p* > 0.05) (Figure 4).

### 3.5. Precision–Recall Curves

Results from the precision–recall analysis, resulting by using a logistic model, show that only in this work, the precision–recall curves (or PR curves) were used because of the presence of unbalanced data. These curves were built by calculating the precision and recall as the classification threshold changes. PR curves show precision on the *y*-axis, against the recall on the *x*-axis. Those quantities are defined as follows:

Precision = TP/(TP + FP)(3)

Recall = TP/(TP + FN)(4)

**Figure 5:**
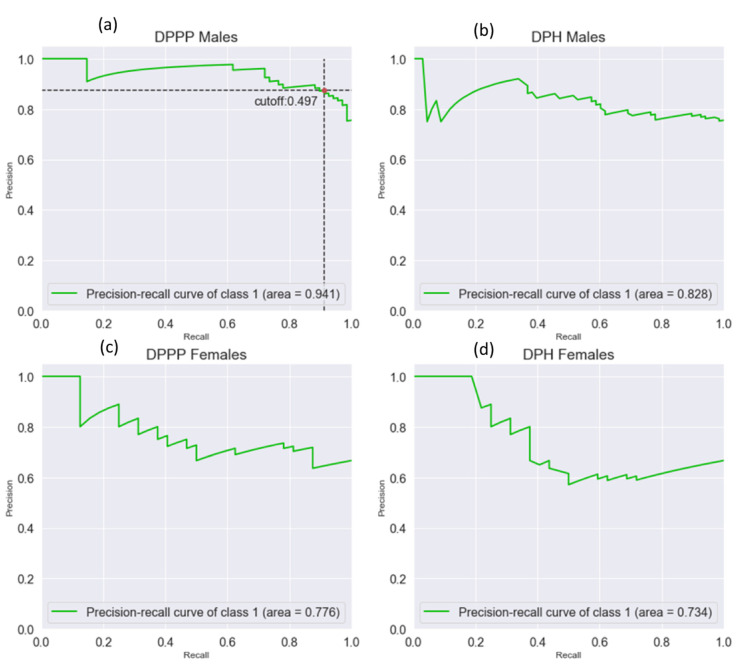
Precision–recall analysis. The four PR curves present the precision–recall analysis. In the insets, the values of the area under the curves are reported. In the DPPP males curve (**a**), the cut-off value of 0.497 is indicated. The precision–recall curve (**a**) highlights that erythrocyte lipid hydroperoxides, measured by DPPP, in men may suggest this parameter as a biomarker to predict CVD in men. The precision–recall curve for DPPP classifier in females (**b**), for DPH classifier in males (**c**), and for DPH classifier in females (**d**).

Where true positive (TP), false positive (FP), and false negative (FN) values were applied. In order to assign a value to those parameters, a cut-off for distinguishing persons between the two classes (controls and CVD) was set. Changing the cut-off allows us to obtain different values for TP, FP, and FN, while the real number of the CVD and controls remains the same. A PR curve shows the relationship between precision and recalls for every possible cut-off value. Thus, every point on the PR curve represents the precision and recall for a chosen cut-off. The best cut-off is the one that allows us to have values of precision and recall both closer to 1. Figure 5 shows the curves for each logistic model. Greater area under the curve and better the classifier performances: this suggests that DPPP Males classifier, with an area under the PR curve of 0.941, is the best performing model among all the classifier that has been used. In this model, the cut-off value that allows to have the highest precision and recall is shown in Figure 5a. It is worth noting that 69 values of the DPH in the original dataset were missing. Those values are almost equally divided in each class (39 CVD and 30 controls). The dataset was imputed by estimating the empirical DPH distribution and using its mean for replacing the missing values.

## 4. Discussion

Our study shows a direct correlation between plasma TMA and erythrocyte lipid hydroperoxides measured by DPPP in men. Plasma TMA in controls is higher in men than in women, but it decreases in male patients with CVD, while it increases in female affected by CVD. TMA production depends mainly on the activity of the gut microflora, which can metabolize dietary choline (phosphatidylcholine, phosphocholine, sphingomyelin, etc.), betaine, and L-carnitine contained in animal food (i.e., meat, fish, egg, etc.). TMA can also be achieved from the catabolism of ergothioneine contained in vegetarian food (i.e., mushrooms, beans). TMA from gut reaches the liver by portal circulation, where flavin monooxygenases (FMO3) catalyze its conversion in the TMAO. Lower expression of FMO3 has been associated with decreased plasma TMAO levels [21,34]. TMAO can also derive from the consumption of fish meals [35]; as observed in mice, it can be produced through the direct oxidation of TMA [22]. TMAO is distributed to tissues, where it works as an osmolyte or can be cleared by the kidneys. It has been suggested that TMAO is involved in the development of atherosclerosis by promoting foam cells formation and atherogenic plaques [36,37]. TMAO contributes to about 11% of the variation in atherosclerosis susceptibility in mice [21], and its level correlates with several characteristics of plaque instability, such as markers of inflammation, platelet activation, and intraplaque hemorrhage [22].

Gender differences in the level of FMO3 have been observed, and a lower TMAO production in the liver of male mice compared to females was measured [21]. The gender difference of TMAO production in mice has also been associated with testosterone level, which is able to downregulate FMO3, and to modulate estrogen-dependent upregulation of FMO3 expression; the testosterone can decrease TMAO levels, and, accordingly, this could explain the lower levels of TMAO in males than in females [38]. Furthermore, an increased excretion rate of TMAO in male mice was measured with respect to female animals when mice were treated with choline; in the same animals, TMA level was higher in male than in female mice. Comparably, in our study, TMA was significantly higher in men than in women; within the group of the same sex, the decreased level of TMA in male CVD patients might be associated with sex-hormone regulation and to its different direct oxidation to TMAO. Females with CVD show higher TMA levels than healthy females, and this might be related to gender differences in the redox system and metabolic responses [11]. Under physiological conditions, women appear to be less susceptible to oxidative stress than men, and this can impact differently on CVD development (i.e., coronary heart disease); gender differences in oxidative stress response might affect the risk of developing atherosclerosis [11]. A link between TMA oxidation and free radicals has been observed; increased TMAO levels have been associated with vascular inflammation through activation of the NLRP3 inflammasome by reactive oxygen species (ROS) due to the TMAO inhibition of the SIRT3-SOD 2-mtROS pathway [39]. ROS production increases in several inflammatory diseases and they have an active role in CVD [40]. In our study, healthy men display higher levels of TMA and lipid hydroperoxides measured by DPPP than male CVD patients. Remarkably, the lipid hydroperoxides in erythrocyte membrane might represent a biomarker of CVD. The precision–recall curves (Figure 5) demonstrate that DPPP can be used to predict CVD only in men. Since our model can discriminate between ill and healthy people, and our goal is to minimize the number of false negatives, only the PR curve of the DPPP males, with an area under the curve of 0.941, can be considered as a possible prognostic factor.

As previously reported, biomarkers of oxidative stress were lower in females than in males [11,41], and healthy women have lower DPPP levels than healthy men. However, in female CVD patients, lipid hydroperoxides increase in respect to males affected by CVD. Changes in the DPPP in men and women could be due also to the double role of ROS, which, in addition to catalyzing the oxidative process, can also have a regulatory function. Lipid hydroperoxides are involved in the stress signaling that mediates several cellular responses (i.e., induction of antioxidant enzymes, apoptotic death) and may have gender-dependent regulations. Furthermore, the different level of hydroperoxides may also be related to differences in the metabolic pathways involved in the removal of lipid hydroperoxides and in the repairing reactions; indeed, lipid hydroperoxides should be removed to avoid the propagation of lipid peroxidation [42]. The excision/reduction/repair pathway comprises the activity of several enzymes like phospholipase A2, glutathione peroxidase (GPX), and an acyltransferase, which, as reported in the kinetic models, are less involved in hydroperoxide repair with respect to the enzymes phospholipid hydroperoxide glutathione peroxidase (PHGPX), phospholipase A2, and acyltransferase, that work through reduction/excision/repair reactions. Transfected cells with PHGPX were particularly efficient in removing lipid hydroperoxides; PHGPH can interact more easily than GPX with polar (i.e., H_2_O_2_) and lower polarity substrates (i.e., lipid hydroperoxides). Gender differences in glutathione peroxidase and phospholipase A2 activity have been observed, and they might contribute to partially explain the gender differences in lipid hydroperoxide levels [11,42,43,44,45].

The increased DPH fluorescence anisotropy in male CVD patients compared to controls indicates a reduced fluidity in the hydrophobic region of the membrane. The decline of membrane fluidity negatively interferes with the membrane protein dynamicity and finally, influences protein functions. Lower fluidity can depend on the fatty acid composition, the oxidative process at lipid or protein level, and metabolic regulation [30,46]. Lipid composition varies according to the diet: a diet rich in mono-polyunsaturated fatty acids (i.e., extra virgin olive oil (EVOO), seed) modulates bilayer composition toward a more fluid state of lipids, compared to a saturated fatty acid diet (i.e., meat, cheese). Oxidative processes decrease membrane fluidity due to the increased number of intermolecular interactions. In healthy subjects, DPH anisotropy was higher in women than in men, and no changes in fluidity were measured in women, even in the presence of CVD. Considering the gender difference observed in lipid hydroperoxides, fluidity changes could mainly depend on dietary lipid composition and metabolic regulation. Changes in lipid profile (i.e., triglycerides, HDL-cholesterol), smoking, and diabetes have a stronger negative impact in post-menopausal women than men, while total cholesterol and LDL have a more significant effect in men [11,47]. Besides, hyperlipidemia and diabetes can amplify oxidative stress, with substantial implications for atherosclerosis development.

The main limitation of this study is the lack of subjects’ dietary habits, including alcohol consumption, to correlate the observed changes at the membrane level. Smoking habits cannot explain observed differences in DPH anisotropy, TMA, and hydroperoxide levels. On the other hand, gender differences measured in the peripheral biomarkers TMA, DPPP, and DPH represent remarkable data, which could be used to better address gender-specific therapies for CVD. Besides, weak changes in TMAO measured in our groups (Supplementary Material Table S1) could be only partially associated with plasma TMA levels. Finally, the direct correlation between plasma TMA and hydroperoxides level that emerged from this study warrants attention, and its potential use as a blood biomarker predictive of CVD in men might have relevant implications in CVD prevention.

Future studies able to correlate gender differences of other biochemical parameters associated with enzymes involved in the regulation of plasma TMA metabolism are warranted, especially considering that recently TMA, but not TMAO, has been proposed as a marker of cardiovascular risk [48,49]. Furthermore, additional studies aimed at the correlation between food components and membrane lipid redox state could be useful to investigate.

## 5. Conclusions

Gender differences in CVD have been identified in plasma TMA level and at the erythrocyte membrane level. Healthy females have a lower basal level of TMA and lipid hydroperoxides than males, while their membrane in the hydrophobic region results in less fluid than in healthy males. Female CVD patients are characterized by a higher plasma TMA than their healthy controls, while no differences in lipid hydroperoxides and membrane fluidity between control and CVD females were measured. Male CVD patients show a decreased fluidity in the hydrophobic region of the erythrocyte membrane. Erythrocyte lipid hydroperoxides measured by DPPP can be proposed as new biomarkers to predict CVD in men.

## Figures and Tables

**Table 1:** Characteristics of the Study Participants.

	Control F = 64/M = 89	CVD F = 125/M = 269
Obesity (BMI ≥ 30)	14/30	46/98
Hypertension	22/41	102/199
Hyperlipidemia	16/24	78/186
Diabetes mellitus	5/16	41/77
Current or past smokers	7/52	43/153

F—females; M—males.

## Supplementary Materials

Supplementary Materials can be found at https://www.mdpi.com/2227-9059/8/8/238/s1.
