# Supplementary material for: Gender-Related Differences in Trimethylamine and Oxidative Blood Biomarkers in Cardiovascular Disease Patients
Source: Biomedicines. 2020 Jul 23;8(8):238. doi: 10.3390/biomedicines8080238 (PMC7460342; doi:10.3390/biomedicines8080238)
Supplement: Supplementary file 1 [file biomedicines-08-00238-s001.pdf]

Table S1. Plasma TMAO level in Male and Female from Control and CVD groups

Gender	Control	CVD
Male	7.82 ± 5.21	8.48 ± 7.23
Female	11.91 ± 11.62	9.51 ± 7.70

$P > 0.05$
